# Complete Genome Sequences of Two JP-I (GI-18) Genotype Infectious Bronchitis Virus Strains Isolated from Chickens with Nephritis in Japan

**DOI:** 10.1128/mra.00156-22

**Published:** 2022-06-23

**Authors:** Masaji Mase, Kanae Hiramatsu, Satoko Watanabe, Hiroshi Iseki

**Affiliations:** a National Institute of Animal Health, National Agriculture and Food Research Organization, Tsukuba, Ibaraki, Japan; b United Graduate School of Veterinary Sciences, Gifu University, Gifu, Gifu, Japan; c Graduate School of Life and Environmental Sciences, Osaka Prefecture University, Izumisano, Osaka, Japan; d Oita Livestock Hygiene Service Center of Oita Prefecture, Oita, Oita, Japan; KU Leuven

## Abstract

We report the complete genome sequences of two strains of JP-1 genotype (GI-18) infectious bronchitis virus (IBV) isolated from the kidneys of dead chickens in Japan in 2000 and 2017. This information will help researchers better understand the evolution and epidemiology of IBV in Japan.

## ANNOUNCEMENT

Avian infectious bronchitis virus (IBV) belongs to the genus *Gammacoronavirus* in the family *Coronaviridae* of the order *Nidovirales* (1). IBV has three major virus-encoded structural proteins, including the spike (S) glycoprotein, the membrane (M) protein, and the nucleocapsid (N) protein. Of these proteins, the spike glycoprotein is formed by the posttranslational cleavage of S1 and S2 polypeptides. The S1 glycoprotein is associated with viral attachment and is a major target of neutralizing antibodies in chickens (2, 3). Additionally, IBV has been typed based on the nucleotide sequence of the S1 region of the spike gene (4–6).

In Japan, seven genotypes of IBV (JP-I, JP-II, JP-III, JP-IV, Mass, Gray, and 4/91) have been confirmed based on the partial nucleotide sequence of the S1 gene (7, 8). Among them, the JP-I genotype was confirmed in strains isolated in the 1960s and has since been prevalent in Japan. Initially, the viruses represented by the JP/KH/64 strain were isolated from chickens with respiratory symptoms (7, 9); since the 1990s, however, virus isolation from dead chickens with nephritis has become prominent (8). In this study, we determined the complete nucleotide sequences of two IBV strains isolated from chickens with nephritis.

The JP/Toyama/2000 strain was isolated from the kidney of a dead chicken in Toyama Prefecture, located in central Japan, in 2000. The JP/Yamagata/2017 strain was isolated from the kidney of a dead chicken in Yamagata Prefecture, located in eastern Japan, in 2017.

These viruses were grown in embryonated chicken eggs and used for genetic analysis at the fourth passage. Viral RNA was extracted from the infected allantoic fluids using a QIAamp viral RNA minikit (Qiagen), and cDNA was synthesized using random hexamer primers (7). As described in previous studies, specific primers for genome sequencing using the Sanger method were used (9–11). Thirty-one pairs of primers were used, and the amplicons were sequenced in both directions. Both the 5′ and 3′ termini of the genome were determined using a rapid amplification of cDNA ends (RACE) kit (Invitrogen).

The sequenced fragments were trimmed and assembled using ATGC-Mac v5 (Genetyx Corp., Japan). The lengths of the complete genomes of the JP/Toyama/2000 and JP/Yamagata/2017 strains, excluding the poly(A) tail, were 27,675 nucleotides (nt), with a G+C content of 38.19%, and 27,667 nt, with a G+C content of 38.15%, respectively. When the open reading frame (ORF) lengths of three JP-I strains in Japan were compared, differences in the lengths of the S, E, M, 4c, and 6b genes were observed (Table 1).

**TABLE 1:** Lengths of ORFs for three strains of IBV genotype JP-I in Japan

ORF	Length (bp) for:		
	JP/KH/64	JP/Toyama/2000	JP/Yamagata/2017
1a	11,859	11,859	11,859
1ab	19,893	19,893	19,893
S	3,510	3,510	3,507
3a	174	174	174
3b	195	195	195
E	324	327	303
M	672	678	669
4b	285	285	285
4c	171	162	162
5a	198	198	198
5b	249	249	249
N	1,230	1,230	1,230
6b	222	222	225

**FIG 1:**
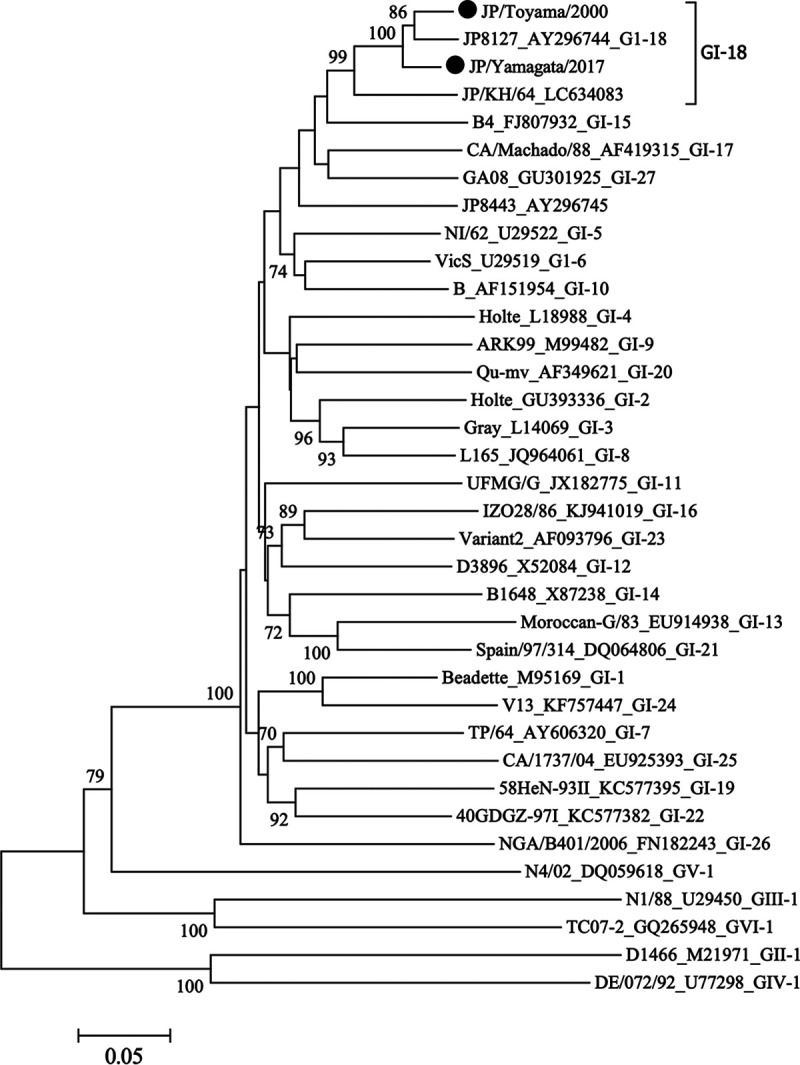
Phylogenetic tree based on the complete S1 glycoprotein gene of IBV. Nucleotides 20368 to 21978 (1,632 bases) of the S1 gene of IBV Beaudette (GenBank accession number NC_001451) (GI-1) were subjected to phylogenetic analysis. The phylogenetic tree was constructed in MEGA 7 (13) using the neighbor-joining method (13) with 1,000 bootstrap replications. Unless otherwise specified, all tools were run with default parameters. Horizontal distances are proportional to the minimum number of nucleotide differences required to connect nodes and sequences. The genotypes of IBV were defined by Valastro et al. (12). The JP/Toyama/2000 and JP/Yamagata/2017 strains are represented by black circles. Other reference strains are listed in the order of strain name, GenBank accession number, and genotype.

Based on the complete nucleotide sequences of S1, IBV was recently classified into 32 genotypes (12). Phylogenetic analysis based on the complete coding sequence of the S1 gene reconfirmed that JP/Toyama/2000 and JP/Yamagata/2017 strains are members of the GI-18 genotype (Fig. 1). The similarity of nucleotide sequences of the S1 gene of the two strains was 94.55%. The information added in this study will help researchers better understand the evolution and epidemiology of IBVs in Japan.

### Data availability.

The genome sequences were deposited in GenBank under the accession numbers LC683779 and LC683780.
